# Renal clearance of endogenous lithium as a marker of proximal tubule function in sepsis and sepsis-associated acute kidney injury: A prospective single-centre study

**DOI:** 10.1186/s12882-026-04743-1

**Published:** 2026-01-08

**Authors:** Kjellbjørn Jakobsen, Hendrik Backmann, Njål Gunnar Nicolaysen, Rasmus Zahn, Sandra Huber, Vegard Skogen, Anders Benjamin Kildal, Lars Marius Ytrebø

**Affiliations:** 1 Anesthesia and Critical Care Research Group, UiT – The Arctic University of Norway, Tromsø, Norway; 2 Department of Anaesthesiology and Intensive Care, Division of Surgery, University Hospital of North Norway, Tromsø, Norway; 3 Department of Laboratory Medicine, Division of Diagnostic Services, University Hospital of North Norway, Tromsø, Norway; 4 Department of Infectious Diseases, Division of Internal Medicine, University Hospital of North Norway, Tromsø, Norway; 5 Department of Clinical Medicine, Faculty of Health Sciences, UiT - The Arctic University of Norway, Tromsø, Norway

**Keywords:** Acute kidney injury, Glomerular filtration rate, Kidney tubules, proximal, Critical illness, Lithium, ICU, Sepsis

## Abstract

**Background:**

Critically ill patients are at high risk of developing acute kidney injury (AKI). Precise functional biomarkers may be useful for monitoring renal tubular function in patients at risk of sepsis-associated AKI (SA-AKI) and to study its pathophysiology in vivo in humans. Lithium is passively reabsorbed in parallel with, and in proportion to, the reabsorption of sodium and water in the proximal tubule. The unreabsorbed fraction is almost entirely excreted in urine, and the fractional excretion of lithium thus provides a semiquantitative estimate of proximal sodium reabsorption. We hypothesized that endogenous lithium reabsorption is impaired in the early stages of SA-AKI. The aim of study was to measure renal clearance of endogenous lithium in patients with SA-AKI and in patients with sepsis without AKI.

**Methods:**

Ten patients with SA-AKI and eight patients with non-AKI sepsis were studied, all without premorbid chronic kidney disease. Blood and urine samples were obtained at 8 AM, 2 PM and 8 PM over the course of a single study day and within the first 48 h after admission to the intensive care unit. Fractional excretion of lithium (FE_Li_) was calculated, and linear mixed-effects model statistics were applied to analyse data.

**Results:**

FE_Li_ was higher in the SA-AKI group compared with the non-AKI group (estimated mean difference 8.26%, *p* = 0.008) with median (interquartile range) FE_Li_ 7.5 (4.6–14.8) % and 3.0 (1.3–4.5) %, respectively. Excluding patients receiving diuretics did not alter the estimated mean FE_Li_ difference (8.79%, *p* = 0.004). Baseline estimated glomerular filtration rate, mean arterial pressure, and peak values for C-reactive protein were similar between groups (*p* = 0.98, 0.43, and 0.52, respectively*)*. SOFA score and peak creatinine concentration during the entire hospital stay were significantly higher in the SA-AKI group (*p* < 0.001).

**Conclusions:**

The results indicate an efficient proximal tubular sodium reabsorption in the sepsis group without AKI, and significantly lower reabsorption in the SA-AKI group. Measurement of endogenous lithium clearance may become useful for studies of proximal tubule function in critically ill patients.

**Trial registration:**

The study was registered at www.clinicaltrials.gov (NCT05982340). Date of registration: 31.7.2023.

## Introduction

Acute kidney injury (AKI) is a common form of organ dysfunction in critically ill patients with sepsis and associated with prolonged hospitalization, requirement for acute dialysis, persistent renal dysfunction, and mortality [1–4]. Sepsis remains the leading cause of AKI in the intensive care unit (ICU) [2, 5–7]. Diagnosis of AKI is currently based on serum creatinine levels and urine output measurements according to the Kidney Disease: Improving Global Outcomes (KDIGO) criteria [8]. However, these measures are known to be unreliable in the perioperative and intensive care settings [1, 9, 10]. Serum creatinine is highly influenced by age, sex, muscle mass, body composition, and does not rise before glomerular filtration rate (GFR) is significantly reduced and time has passed to allow the buildup of creatinine in serum [1, 11, 12]. Moreover, experimental data suggest that creatinine generation may be reduced in sepsis [13]. Significant renal impairment may thus develop before the KDIGO criteria for AKI are fulfilled.

Several biomarkers of renal and tubular damage have been suggested as supplemental diagnostic and research tools [3], and some are also proposed to be included in the definition of AKI [14]. However, histopathological alterations in sepsis-associated AKI (SA-AKI) are frequently less pronounced than the accompanying decline in GFR, indicating that renal functional impairment may exceed what is evident from structural damage alone [15, 16]. Furthermore, unlike markers of GFR, these damage biomarkers do not provide real-time assessment of renal function but merely indicate that structural injury has occurred.

Functional biomarkers may provide distinct insight into pathophysiological processes that differ from those revealed by damage biomarkers. By providing mechanistic data they may become valuable research tools for the study of SA-AKI. Apart from markers of GFR, few other functional markers of kidney function have been explored in clinical studies, even less so in AKI. Limiting the assessment of renal function to filtration alone represents a major limitation of currently available non-invasive methods for evaluating renal function. In particular, greater attention should be directed towards the reabsorptive role of the proximal tubules, as this function accounts for a substantial proportion of the renal energy and oxygen consumption [17]. Molecules that are freely filtrated at the glomerulus and completely reabsorbed by the proximal tubules are the ideal functional proximal tubule biomarkers [18].

In the search for potential biomarkers to assess proximal renal tubule function in critically ill patients, lithium emerged as a particularly interesting candidate. Lithium is an alkali metal found in trace substances in human blood [19]. It is not bound to plasma proteins, thus it passes the glomerular membrane in its entirety [20]. Lithium itself is not actively transported; instead, it is passively reabsorbed in parallel with sodium and water in the proximal tubule, in proportion to their proximal tubular reabsorption [20]. The fraction that is not reabsorbed in the proximal tubule is known to be excreted in urine almost in its entirety, thereby enabling estimation of the proximal tubular sodium reabsorption through calculation of the fractional excretion of lithium (FE_Li_) [20–22]. The proportion of sodium reabsorbed in the proximal tubule (R_Naprox_) is commonly expressed as a percentage of the filtrated load and can be derived from the relationship *R*_*Naprox*_*=1-FE*_*Li*_ [23]. The first attempts to estimate sodium reabsorption using lithium were made by administering exogenous lithium to reach detectable concentrations in blood [22]. Following the development of new laboratory methods, measurement of endogenous lithium in blood and urine became clinically feasible [22].

We hypothesized that proximal tubule function as estimated by endogenous lithium clearance is impaired in the early stages of SA-AKI. The aim for study was to measure endogenous lithium renal clearance in patients with SA-AKI and in patients with sepsis without AKI.

## Methods

This study was approved by the Regional Ethical Committee of North Norway (record 536547/2022) and the Institutional Board at the University Hospital of North Norway (record ID 3055). The study was registered at www.clinicaltrials.gov (NCT05982340) and performed at the University Hospital of North Norway. The STROBE guidelines for observational studies have been followed when reporting the study [24].

### Power calculation

Steinhäuslin et al. observed FE_Li_ in nine individuals with acute tubular necrosis (mean age 60) to be 26.1% (mean R_Naprox_ = 73.9%) [25]. Fliser et al. has studied R_Naprox_ in 29 healthy elderly (mean age 68) and found a mean R_Naprox_ of 87% (SD = ± 6) corresponding to an FE_Li_ of 13% [26]. Sample size calculations were performed using a two-sided independent samples t-test. Based on the data from Fliser and Steinhäuslin et al. a difference of 13.1% was assumed. Standard deviation from Steinhäuslins data is not presented and is therefore assumed to be equal to data from Fliser et al. Applying a significance level of 0.05 and a power of 90%, the required sample size was calculated to be ≈ 6 per group. Recruitment of 10 individuals for each group was deemed appropriate, which accounts for assumptions in the power calculation as well as dropouts.

### Study design

Data was collected from June 2024 through February 2025, and the study was performed in the ICU at the University Hospital of North Norway. ICU patients with sepsis diagnosed or suspected were included to establish two groups of individuals, 10 individuals with SA-AKI and 10 without AKI. SA-AKI was defined according to the 28th ADQI consensus criteria [27]. Suspicion of sepsis was defined as qSOFA ≥ 2/3 in combination with infection, or strong clinical suspicion of sepsis. If not already performed, sepsis was later defined by a SOFA score of ≥ 2 due to infection, as per Sepsis-3 recommendations [28]. Baseline SOFA score was assumed to be zero in patients without known pre-existing organ dysfunction. Patients included based on suspicion of sepsis were excluded unless the diagnosis of sepsis was subsequently made. AKI was defined according to the Kidney Disease: Improving Global Outcomes (KDIGO) criteria for AKI [8]. Only the serum creatinine component of the definition was applied; urine output criteria were excluded from the classification as we did not expect precise urine output data to be consistently available across all participants at inclusion. Individuals with AKI onset more than 48 h before possible inclusion were excluded to preserve group homogeneity, as participants were enrolled on the first day after admission to the ICU. Exclusion criteria were postrenal AKI, suspicion of or clinically confirmed urinary infection as well as preexisting CKD as determined by KDIGO criteria [29]. Estimated GFR (eGFR) 60–90 mL/min/1.73m^2^ was not considered indicative or diagnostic of CKD unless accompanied by other markers of kidney damage, in accordance with the KDIGO criteria. For patients without pre-existing data on renal function, we evaluated the observed acute rise in serum creatinine. Patients were included if the observed increase would meet AKI criteria regardless of the assumed baseline, provided it lay within the normal reference range. A baseline creatinine was later imputed through backwards calculation using the 2009 CKD-EPI Creatinine formula, assuming a baseline eGFR of 75 mL/min/1.73m^2^ [8, 30]. This was then compared to the lowest creatinine measured prior to hospital discharge, and the lower value of the two was ultimately applied as the baseline value.

Inclusion was halted groupwise when the target was met. AKI is a dynamic condition, and swift inclusion was necessary due to the nature of the research. As such, a planned survey of patient data and blood work up following inclusion of all 20 individuals was executed to ensure valid inclusion in the study as well as correct group allocation. This survey was performed prior to performing calculations or statistical analysis.

### Experimental procedure

All participants were included on the first day after admission to the ICU. Blood and urine samples were obtained at 8 AM, 2 PM and 8 PM over the course of a single study day and within the first 48 h of ICU admission. Blood was obtained from a radial artery catheter. 5 ml of blood was collected at each sampling point in certified Vacutainer tubes for trace element determination (*BD Vacutainer ref 368380*). Vacutainers were placed on the bench for one hour for coagulation and subsequently centrifuged for 10 min with 1300 G at 20 °C. 1 ml aliquots of serum were pipetted into three Sarstedt Screw cap 2 ml micro cryo-tubes (Sarstedt AG & Co, Nümbrecht, Germany) and stored at -70 °C for later analyses.

Urine was obtained from a bladder catheter by connecting a sterile urine bag to the catheter one hour before each sampling time. Time passed in minutes was recorded for improved accuracy. Urine volume from each participant was measured and 1 ml aliquots of urine were pipetted into three Sarstedt Screw cap 2 ml micro cryo-tubes (Sarstedt AG & Co, Nümbrecht, Germany) and stored at -70 °C for later analyses. Routine laboratory analyses were performed at the Department of Laboratory Medicine, University Hospital of North Norway, Tromsø, and clinical values, such as mean arterial pressure (MAP), were measured at 8:00 AM of the study day.

### Determination of lithium in serum and urine

The lithium analyses were performed at the Environmental Pollutant Laboratory, Department of Laboratory Medicine, University Hospital of North Norway, applying inductively coupled plasma mass spectrometry (ICP-MS) as described previously [31]. Sample preparation was conducted by an automated liquid handler (Tecan Freedom Evo 200, Männedorf, Switzerland) by dilution of either 100 µL serum or urine with a solution consisting of Milli-Q water (Millipore/Merck KGaA, Darmstadt, Germany), 10% v/v ammonia (Honeywell Fluka, Bucharest, Romania) and 2-propanol (Honeywell Fluka, Bucharest, Romania), 0.08% v/v Triton X-100 (Sigma/ Merck KGaA, Darmstadt, Germany) and 0.25 µg/L gold standard (Au; Inorganic Ventures, Christiansburg, VA, USA) followed by mixing on a shaker. A NEXION 300D ICP-MS system (Perkin Elmer, Waltham, Massachusetts, USA) equipped with an ESI-Fast SC2DX auto sampler was used for instrumental analysis where an internal standard solution was introduced via a T-piece containing 20 µg/L rhodium (Rh^103^; Inorganic Ventures, Christiansburg, VA, USA). For the MS analysis the kinetic-energy-discrimination mode with helium as reaction gas was applied and measurements were conducted in triplicates. A matrix matched calibration curve with ClinCal serum or ClinCal urine calibration material from Recipe (Recipe, Munich, Germany) was used for quantitative determination of lithium in the samples. Samples were analysed batchwise with each batch containing 32 samples, three ClinCal calibration samples diluted 1:100, 1:40 and 1:20 respectively, one calibration blank sample, four blank samples and two sets of ClinCheck control material level 1 and level 2 from Recipe (Recipe, Munich, Germany), and Seronorm level 1 and level 2 (Sero, Billingstad, Norway) for quality assurance and quality control. Diluent blanks for control of the background and instrumental carry over were also included. Additionally, the laboratory participates successfully in the international quality control program Quebec Multielement External Quality Assessment Scheme (QMEQAS) organized by the Centre de Toxicologie du Quebec, Quebec, Canada, which also covers lithium analysis in human urine. All the equipment used for sample collection was tested for lithium contamination and prior application and was found to be free from lithium contribution and therefore suitable for the present study.

### Calculations

Glomerular filtration rate was estimated using the 2009 CKD-EPI Creatinine formula [30]. Clearance of lithium (C_Li_) and creatinine (C_Cr_) were calculated by the following formula:

$${C_x}={\text{ }}V{\text{ }}x{\text{ }}{U_x}/{P_x}$$

Where x is lithium or creatinine. V was the urinary flow rate in millilitres per minute and U_x_ and P_x_ were the concentration of the solutes in urine and serum, respectively.

The fractional excretion of lithium (FE_Li_) was calculated by the use of the formula: FE_Li_ = C_Li_ / C_Cr_ [23]. Estimated fractional proximal sodium reabsorption (R_Naprox_) was calculated by using the formula: R_Naprox_ = 100% – FE_Li_ % [23]. The fractional excretion of sodium (FE_Na_) was calculated by the use of the formula: FE_Na_ = (U_Na_ x P_Cr_)/(P_Na_ x U_Cr_) x 100 [32].

### Statistics

All statistical analyses were conducted using Stata (MP18.0 for Mac, StataCorp LLC). Linear mixed-effects model was applied to assess overall differences in FE_Li_, R_Naprox_, FE_Na_ and creatinine clearance (CrCl) between participants with and without SA-AKI. To evaluate the potential impact of diuretic use on the primary outcomes, additional mixed-effects regression models were performed after excluding participants who received loop diuretics during the study period (*n* = 2). To assess whether age could confound the association between group status and FE_Li_, we repeated the mixed-effects regression analyses including age as a covariate. To assess whether the effect of group differed across sampling times, we fitted two nested mixed-effects models. The first model included only the main effects of group and sampling time, while the extended model additionally included their interaction. The two models were then compared using a likelihood-ratio test, with the simpler model nested within the extended model.

Model assumptions and normality in both raw data and residuals were assessed visually using histograms and Q-Q plots and residual-versus-fitted plots, and robust standard errors were applied to ensure valid inference. Continuous variables were described using medians and interquartile ranges (IQR) due to non-normal distribution of the data, and categorical variables were presented as counts and percentages. No imputation was performed for missing values.

Comparisons of demographic data between the groups were conducted using Wilcoxon rank-sum test for continuous and ordinal variables due to non-normal distribution and small group sizes. Fisher’s exact test was used for binary categorical variables when expected frequencies were low. Exact *p*-values were reported, and *p* < 0.05 was considered significant.

## Results

20 patients were recruited and there were no dropouts during the study day. Two individuals originally allocated to the non-AKI group were excluded prior to data analysis. One patient was excluded as the discharge diagnosis proved to be urosepsis, which was an exclusion criterion in the study protocol. The second patient was retrospectively found to have developed AKI during same course of illness, but with onset > 48 h before inclusion. The remaining patients were all correctly allocated at the time of inclusion. Thus, ten patients with SA-AKI and eight patients with non-AKI sepsis were enrolled for calculations and statistical analyses. Baseline creatinine was available for all patients except two patients in the SA-AKI group. Imputation of baseline creatinine was performed but was found to give a higher baseline creatinine than the lowest pre-discharge creatinine value for both patients.

Patient demographics is presented in Table 1. The median age was significantly lower in the SA-AKI group compared to the non-AKI group (61.5 vs. 74.5 years, *p* = 0.043). Sex distribution did not differ significantly between groups. SOFA scores were significantly higher in the SA-AKI group (median 9 vs. 3.5, *p* < 0.001). Peak values for C-reactive protein during the entire hospital stay was similar between groups (252 vs. 234 mg/L, *p* = 0.52). Median AKI stage in the SA-AKI group was 2, with all three stages being represented in the population. Peak creatinine concentrations during the entire hospital stay were significantly higher in the SA-AKI group (median 255 vs. 83.5 µmol/L, *p* < 0.001), whereas median baseline eGFR did not differ (86.5 vs. 85 mL/min/1,73 m^2^, *p* = 0.98). There was no significant difference in MAP between groups (73 vs. 75 mmHg, *p* = 0.43). However, the use of vasoactive drugs was significantly more common in SA-AKI patients. Nine out of ten SA-AKI patients received noradrenaline, which was the only vasoactive drug administered to any of the study participants. Neither 24-hour urine output volume nor use of diuretics differed significantly between groups.

**Table 1:** Demographics of the study participants

Variable	SA-AKI (*N* = 10)	Non-AKI (*N* = 8)	*p*-value
Age, years	61.5 (23–79)	74.5 (38–82)	0.04^9^
Male sex (%)	50%	88%	0.15^10^
SAPS II score^1^	49.5 (22–60)	32.5 (16–48)	0.18^9^
SOFA score^2^	9 (5–11)	3.5 (1–8)	< 0.001^9^
AKI stage (0–3)^3^	2 (1–3)	0 (0–0)	< 0.001^9^
Peak CRP (mg/L)	252 (153–377)	234 (90–348)	0.52^9^
Peak creatinine (µmol/L)	255 (92–420)	83.5 (63–97)	< 0.001^9^
Baseline eGFR^4^, (mL/min/1,73 m^2^)	86.5 (62–102)	85 (65–113)	0.98^9^
MAP (mmHg)^5^	73 (65–95)	75 (65–103)	0.43^9^
Vasoactive medication (% of patients)^6^	90%	25%	0.01^10^
Diuretics (% of patients)^7^	0%	25%	0.18^10^
Diuresis (mL/24h)^8^	2358 (1065–4800)	2278 (937–3757)	0.76^9^
Intensive care unit mortality	10%	0%	1.00^10^
In-hospital mortality	10%	0%	1.00^10^
Renal replacement therapy	0%	0%	N/A

Presented data are median values and in parenthesis the range, unless otherwise specified in the first column. Data is presented for participants with sepsis-associated acute kidney injury (SA-AKI) and sepsis without AKI (Non-AKI), respectively

^1^ Simplified Acute Physiology Score II [42]

^2^ Sequential Organ Failure Assessment [28]

^3^ Acute Kidney Injury Stage, as per KDIGO criteria [8]

^4^ 2009 CKD-EPI formula [30]

^5^ Mean arterial pressure (MAP) was measured at 8:00 AM on the study day

^6^% of patients that had received vasoactive medication during the study day

^7^% of patients that had received diuretics during the study day

^8^ Urine output from 7 AM on the study day to 7 AM the following day

^9^ Wilcoxon rank-sum (Mann-Whitney) test

^10^ Fisher’s exact test

**Fig. 1:**
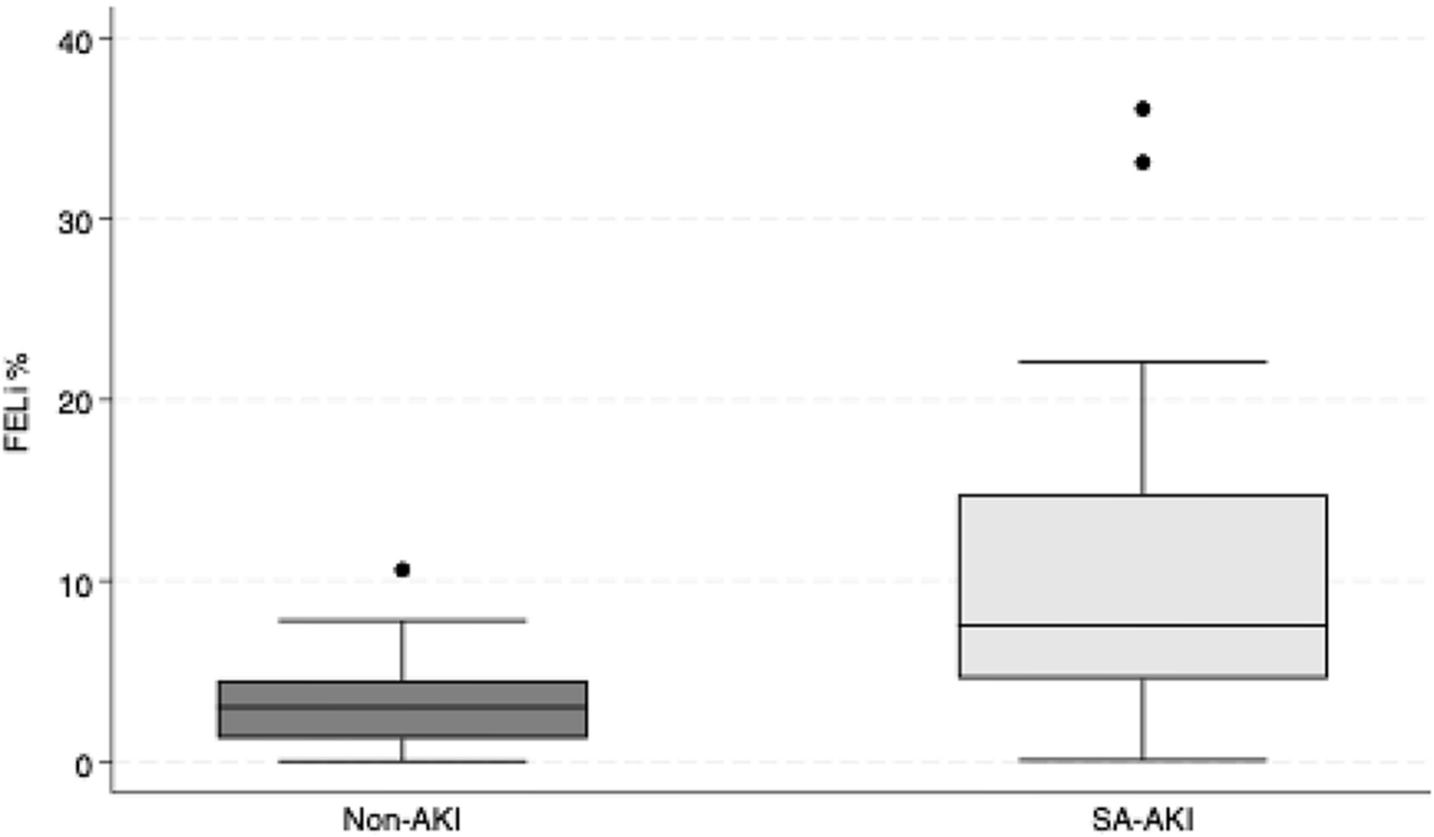
Boxplot FE_Li._ The boxplot illustrates fractional excretion of lithium (FE_Li_, %) in participants with sepsis-associated acute kidney injury (SA-AKI, *n* = 10; 28 observations) and sepsis without AKI (Non-AKI, *n* = 8; 19 observations)

Results from the mixed-effects regressions are presented in Table 2, descriptive statistics are presented in Table 3 and the distribution of FE_Li_ across groups is illustrated in Fig. 1. Median FE_Li_ was 3.0% in the non-AKI group and 7.5% in the SA-AKI group. FE_Li_ was significantly higher in the SA-AKI group compared with the non-AKI group, with an estimated mean difference of 8.26% (95% CI: 2.15% to 14.37%, *p* = 0.008), corresponding to a mean difference in R_Naprox_ of -8.26%. The intraclass correlation coefficient (ICC) was 0.73, indicating that 73% of the total variability in FE_Li_ was attributable to differences between participants rather than within-participant changes over time. When excluding participants receiving diuretics, the estimated group difference in FE_Li_ remained essentially unchanged at 8.79% (95% CI: 2.80% to 14.79%, *p* = 0.004). Similarly, adjustment for age did not meaningfully change the group difference in FE_Li_ (coefficient 9.36%, 95% CI 1.37%-17.35%, *p* = 0.022). Age itself was not significantly associated with FE_Li_ (*p* = 0.545). Examination of whether the effect of group changed across sampling times was performed by comparing a model with main effects only to an extended model including an interaction between them. Inclusion of the interaction did not improve the model fit (*p* = 0.34).

**Table 2:** Results of mixed-effects regression models for renal proximal tubule function markers and glomerular filtration marker

Variable	Dataset	Intercept Non-AKI group (95% CI)	Coefficient for SA-AKI vs. Non-AKI (95% CI)	*p*
FE_Li_ (%)^1^	All patients (*N* = 18)	3.61 (1.76–5.47)	8.26 (2.15–14.37)	0.008
	Excluding patients on diuretics (*N* = 16)	3.08 (1.69–4.45)	8.79 (2.80–14.79)	0.004
R_Naprox_ (%)^2^	All patients (*N* = 18)	96.37 (94.54–98.24)	-8.26 (-14.37 - -2.15)	0.008
CrCl (mL/min)^3^	All patients (*N* = 18)	66.73 (48.59–84.87)	-24.64 (-55.11–5.84)	0.113
	Excluding patients on diuretics (*N* = 16)	69.88 (49.08–90.68)	-27.79 (-59.98–4.41)	0.091
FE_Na_ (%)^4^	All patients (*N* = 18)	1.46 (-0.03–2.94)	0.84 (-1.19–2.86)	0.418
	Excluding patients on diuretics (*N* = 16)	0.57 (0.26–0.87)	1.65 (0.28–3.02)	0.018

Results of mixed-effects regression with two groups; patients with sepsis-associated acute kidney injury (SA-AKI) and sepsis without acute kidney injury (Non-AKI). Intercept represents the estimated mean in the Non-AKI group (reference). Group difference corresponds to the coefficient for SA-AKI vs. non-AKI. p-values correspond to the fixed effect of group in the mixed-effects regression model

^1^ Fractional excretion of lithium

^2^ Estimated fractional proximal sodium reabsorption

^3^ Creatinine clearance

^4^ Fractional excretion of sodium

**Table 3:** Descriptive statistics for renal proximal tubule function markers and glomerular filtration marker

Variable	Group	Observations	Median (IQR)	Mean ± SD	Range
FE_Li_ (%)^1^	Non-AKI	19	3.01 (1.29–4.50)	3.56 ± 2.79	0.02–10.6
	SA-AKI	28	7.53 (4.59–14.78)	10.62 ± 9.03	0.08–36.1
R_Naprox_ (%)^2^	Non-AKI	19	97.0 (95.5–98.7)	96.4 ± 2.8	89.4–99.98
	SA-AKI	28	92.5 (85.2–95.4)	89.4 ± 9.0	63.9–99.9
CrCl (mL/min)^3^	Non-AKI	20	66.0 (37.9–87.0)	66.0 ± 37.7	7.5–167.6
	SA-AKI	28	25.0 (11.2–70.8)	43.2 ± 41.7	3.7–142.0
FE_Na_ (%)^4^	Non-AKI	20	0.54 (0.26–1.54)	1.38 ± 2.30	0.08–9.89
	SA-AKI	28	0.48 (0.29–2.81)	2.18 ± 3.25	0.08–12.3

Descriptive statistics for the two groups; patients with sepsis-associated acute kidney injury (SA-AKI) and sepsis without acute kidney injury (Non-AKI). Data are presented as median with interquartile range, mean with standard deviation, and range

^1^ Fractional excretion of lithium

^2^ Estimated fractional proximal sodium reabsorption

^3^ Creatinine clearance

^4^ Fractional excretion of sodium

**Fig. 2:**
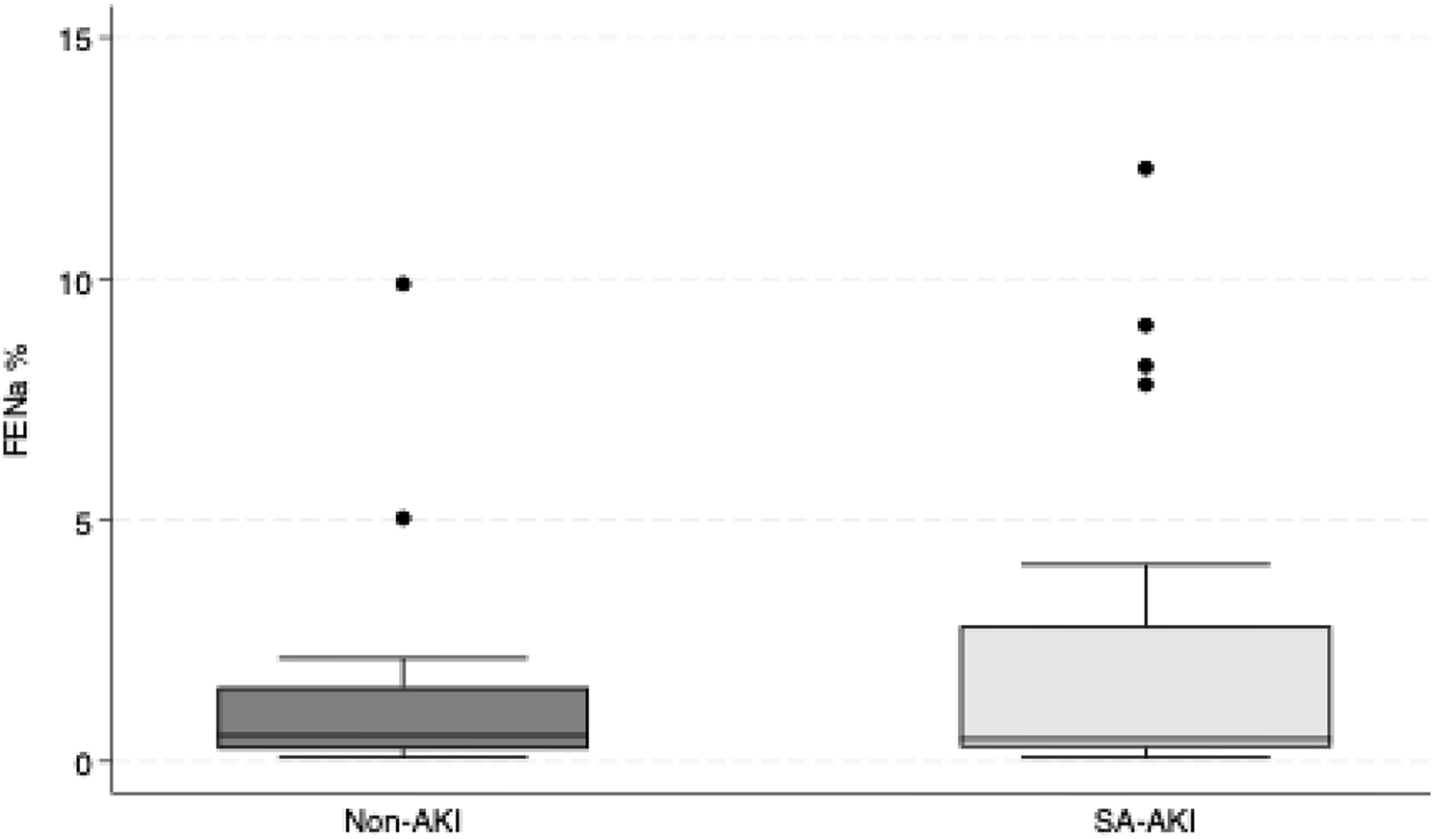
Boxplot FE_Na_. The boxplot illustrates fractional excretion of sodium (FE_Na_, %) in participants with sepsis-associated acute kidney injury (SA-AKI, *n* = 10; 28 observations) and sepsis without AKI (Non-AKI, *n* = 8; 20 observations)

In contrast to FE_Li_, FE_Na_ did not differ significantly between groups (mean difference: 0.84%, 95% CI: −1.19% to 2.86%, *p* = 0.418). However, when excluding participants receiving diuretics, the group difference became significant, with the SA-AKI group showing 1.65% higher FE_Na_ (95% CI: 0.28% to 3.02%, *p* = 0.018). Figure 2 shows the distribution of FE_Na_ in participants with and without SA-AKI.

**Fig. 3:**
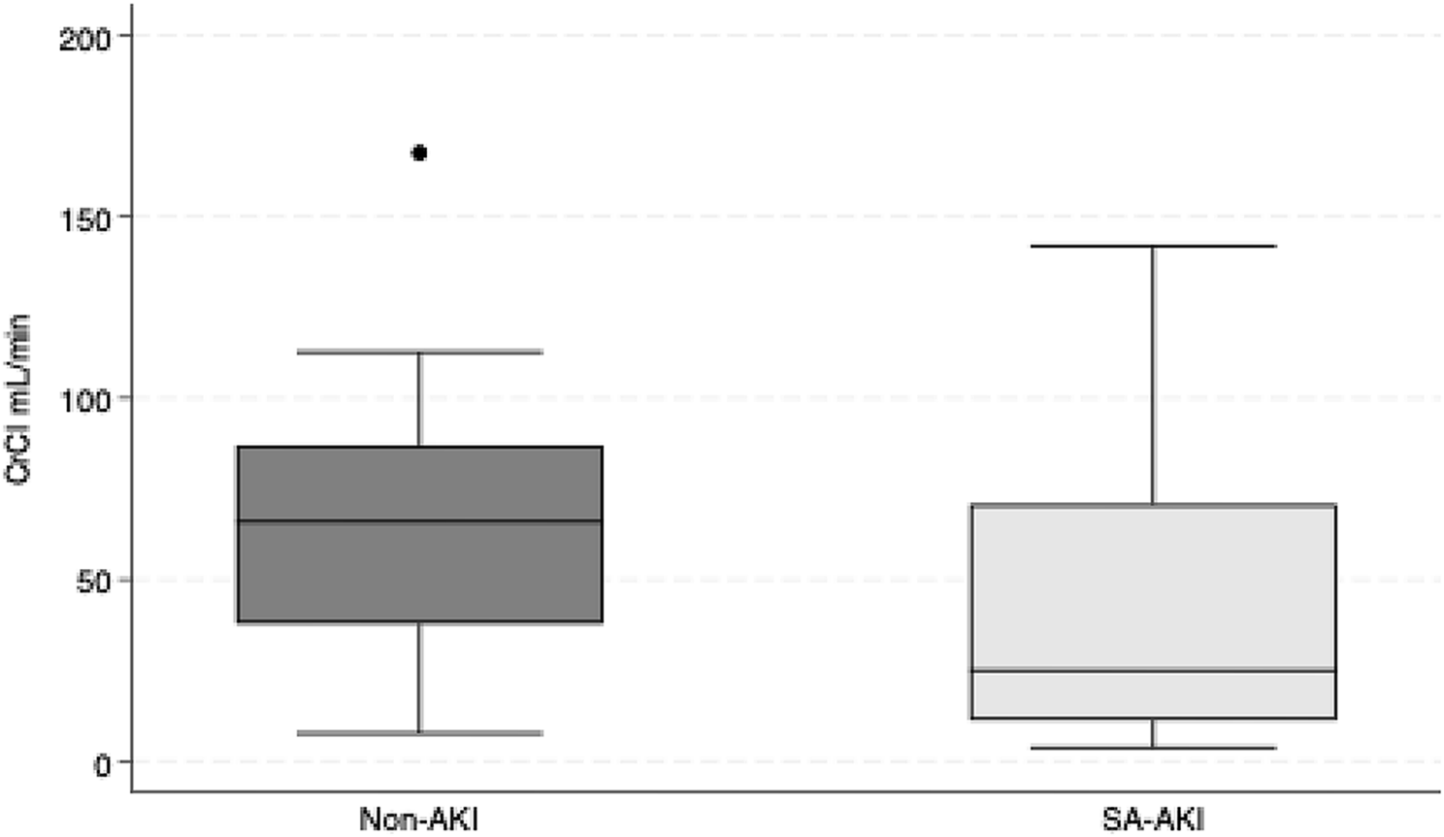
Boxplot CrCl. The boxplot illustrates creatinine clearance (CrCl, mL/min) in participants with sepsis-associated acute kidney injury (SA-AKI, *n* = 10; 28 observations) and sepsis without AKI (Non-AKI, *n* = 8; 20 observations)

There was no significant difference in mean CrCl between the groups (mean difference: -24.64 mL/min, 95% CI: -55.11 to 5.84, *p* = 0.113). The distribution of CrCl between the two groups is shown in Fig. 3.

## Discussion

Monitoring of tubular reabsorption in patients with SA-AKI may provide valuable information beyond the traditional measures. The present study demonstrates two distinct patterns; non-AKI patients exhibit efficient proximal tubular sodium reabsorption (median R_Naprox_ 97.0%), whereas patients with SA-AKI show significantly lower proximal tubular sodium reabsorption as determined by FE_Li_ (median R_Naprox_ 92.5%) (Tables 2 and 3. Figure 1). When excluding participants receiving diuretics, the estimated group difference remained essentially unchanged (Table 2). These findings suggest a robust association between SA-AKI and elevated FE_Li_, which are independent of diuretics.

FE_Na_ did not differ significantly between the groups; however, significance was reached after exclusion of diuretic-treated patients (Table 2). Importantly, baseline renal function, as estimated by eGFR, was similar between the two groups (*p* = 0.98, Table 1). This strengthens the representativeness and comparability of the two populations and give support to the interpretation that differences in FE_Li_ are related to AKI itself, rather than pre-existing renal impairment. Notably, the marked difference in peak creatinine between the groups during the hospital stay further strengthens the validity of the group classification, as it reflects the full course of renal function during admission (Table 1).

The data are consistent with the interpretation that FE_Li_ may reflect underlying mechanisms of kidney injury. Fliser et al. reported a mean FE_Li_ of 13% in healthy individuals of similar age range as the patients included in the current study [26]. Both groups appear to have lower FE_Li_ values than the healthy individuals in the study by Fliser et al. (non-AKI median FE_Li_ 3.0%, SA-AKI median FE_Li_ 7.5%, Table 3). Accordingly, the current findings suggest a markedly increased sodium reabsorption in both study groups, though ultimately of significantly greater magnitude in the non-AKI group.

The relationship between GFR and tubule function is fundamental in order to understand renal function [18, 33]. The low median FE_Li_ observed in both groups, particularly in the non-AKI group, indicates an effective increase in sodium reabsorption. This is in line with findings from prior studies in humans and sheep, where FE_Na_ has been demonstrated to decrease in sepsis [34, 35]. However, the overall higher FE_Li_ in the SA-AKI group, together with a wider distribution of FE_Li_, suggest not only a reduced ability to sustain this response, but also the presence of overt tubular dysfunction leading to inappropriate sodium losses. One potential underlying mechanism for such dysfunction may be bioenergetic failure of the proximal tubule, where limited ATP availability compromises Na^+^/K^+^-ATPase activity and thereby reduces sodium reabsorptive capacity. The reduced GFR in the SA-AKI group could represent a compensatory mechanism to limit sodium excretion and thus preserve intravascular volume in the context of tubular dysfunction [33, 36]. The macula densa regulates the renin-angiotensin-aldosterone system (RAAS) by sensing sodium chloride concentration in the filtrate of the proximal distal convoluted tubule [37]. A high sodium chloride concentration stimulates RAAS and in turn suppresses GFR. As the majority of sodium reabsorptive capacity is provided by the proximal tubule [37], a high sodium concentration in the distal tubule necessitates a compensatory fall in GFR. The RAAS mediated reduction lowers the total filtered sodium load, thus preventing excessive natriuresis. Therefore, a marked reduction in GFR serves as a vital compensatory mechanism to preserve intravascular volume. Reduced GFR and increased FE_Li_ may suggest that this mechanism is activated in certain critically ill patients suffering from SA-AKI.

While lithium reabsorption is the most precise non-invasive marker of sodium handling in the proximal tubule, the method has certain limitations. Evidence suggests that the fractional delivery of lithium slightly exceeds that of water at the end of the proximal convoluted tubule [38]. In other words, the lithium reabsorption is not perfectly proportional to reabsorption of water, it occurs at a modestly lower rate than water reabsorption. Conversely, a small degree of lithium reabsorption is thought to occur in the loop of Henle, without parallel reabsorption of water [20, 38]. These two opposing sources of error counterbalance each other, thereby reducing their overall impact on the utility of FE_Li_ as a marker of proximal tubular sodium and water reabsorption [39]. Nevertheless, these imperfections introduce a degree of inaccuracy in the estimation of proximal sodium reabsorption. Accordingly, some authors have proposed that FE_Li_ should be used primarily as a directional marker of alterations in proximal tubule reabsorption [38]. The most recent review, however, argue for its interpretation as a semiquantitative measure [39].

FE_Li_ is known to increase with the use of loop diuretics, most likely due to inhibition of reabsorption of lithium from loop of Henle and reduced reabsorption of lithium in the proximal tubule [20]. In the present study, none of the patients in the SA-AKI group received diuretics on the study day. In contrast, two patients in the non-AKI group received diuretics, exclusively the loop diuretic furosemide. One of those two patients had the highest FE_Li_ among the non-AKI patients, while the other patient had a value above the group median. Despite this, the group-level finding remained: FE_Li_ was significantly lower in the non-AKI group. We believe the inclusion of these patients is justified and does not undermine the overall conclusion. The decision to include patients receiving diuretics was partly based on the clinical expectation that some would require such treatment after inclusion and initial blood and urine sampling, and partly to enhance the external validity of the study. Nevertheless, inclusion of patients who received diuretics limits the interpretability of FE_Li_ as a pure measure of proximal sodium reabsorption. To address this, we performed additional analyses restricted to patients not receiving diuretics. These analyses confirmed that the group difference in FE_Li_ not only persisted but slightly increased when diuretic users were excluded (Table 2). Though we sought to examine the impact of diuretic use on the relationship between FE_Li_ and SA-AKI, ultimately our cohort only had 2 diuretic-treated patients; though we did not detect any influence on diuretic use in our cohort, that may have been due to limited sample size.

Interestingly, FE_Na_ showed no significant difference between the two groups, but was significantly different when the two diuretic-treated patients were excluded (Table 2). FE_Na_ is commonly used in clinical practice as a helpful tool to distinguish prerenal from intrinsic renal causes of AKI, but Steinhäuslin has demonstrated that FE_Li_ may be a better determinator in this regard [25]. Despite differences in study populations, Steinhäuslin et al. examined FE_Li_ in acute tubular necrosis, prerenal failure, and healthy controls, our data support the potential value of FE_Li_ in the diagnostic evaluation of critically ill patients. Moreover, FE_Li_ may be more robust than FE_Na_ in patients treated with diuretics, yet this observation should be further investigated. FE_Na_ has been studied in experimental models of SA-AKI, and has been shown to significantly decrease over time despite an increase in renal blood flow [34]. These findings indicate that FE_Na_ has limited ability to distinguish between pre-renal and intrinsic AKI in the context of sepsis.

Certain limiting factors challenge the validity of the current study. The SA-AKI and sepsis without AKI group were not age matched. Participants in the SA-AKI group were, on average, younger than those in the non-AKI group (Table 1). Age is a known confounder of lithium clearance; however, the magnitude of this difference is unlikely to explain the gap in FE_Li_ between the study groups. Fliser et al. studied the difference in young healthy individuals (mean age 26 years) and elderly healthy individuals (mean age 68 years) and found FE_Li_ to differ by 10%, with the lowest FE_Li_ being among the healthy elderly [26]. The absolute difference in median age between the groups in our study was 13 years, a mere third of the difference in Flisers study. Age was ultimately not significantly associated with FE_Li_ in our study, and adjusting for age did not attenuate the difference between the SA-AKI and non-AKI groups. This suggests that the observed group difference in FE_Li_ is unlikely to be explained by age alone. A possible explanation of the age discrepancy is that older individuals in the non-AKI group may have developed less severe systemic illness yet still met criteria for critical care unit admission and sepsis. This might be due to lower physiological reserve in the elderly.

Time was not found to be a significant factor for FE_Li_ in the present study. However, the current study was not designed with sufficient power to uncover such differences, and only a short time course of illness was observed for each individual. A known confounder for urinary clearance measurements is inadequate urine collection, but this was negligible in the current study as the urine was collected and processed by dedicated and well-trained study personnel using appropriate collection equipment and stopwatch for accurate timing. Another weakness of the current study is the use of creatinine as a marker of GFR. The KDIGO AKI criteria has previously been demonstrated to be inaccurate at diagnosing AKI compared to continuous measurement of GFR using iohexol [40]. Accordingly, future studies on the relationship between glomerular filtration and proximal tubule function should utilize the more accurate continuous iohexol infusion method [40, 41].

The descriptive analyses indicated several clinically relevant differences between patients with and without SA-AKI. The higher SOFA scores and more frequent vasopressor use among those with SA-AKI point to greater overall disease severity and hemodynamic instability (Table 1). The absence of significant differences in MAP likely reflects effective hemodynamic support, while similar CRP levels suggest that the degree of systemic inflammation alone may not fully explain the observed differences. The high urine output, most noteworthy in the SA-AKI group, may indicate impaired tubular sodium reabsorption and a compensatory response to maintain volume balance rather than preserved renal function.

This study is limited by a small sample size, which reduces the precision of the observed effect estimates and limits the ability to perform valid subgroup analyses in potentially heterogeneous populations. Although the sample size was determined by an a priori sample size calculation; the findings should be interpreted cautiously. In addition, the single-centre design limits the external validity and generalizability of the results, and confirmation in larger, multicentre studies is warranted.

We excluded individuals with suspected or clinically confirmed urinary tract infections. To our knowledge, renal lithium handling has not previously been studied in this context. Because urinary infections may be associated with renal inflammation or nephritic abscesses, we chose to exclude these patients to reduce the risk of confounding effects on proximal tubular function unrelated to the pathophysiological mechanisms of sepsis itself. The role of FE_Li_ in this patient population should be explored in future studies.

A range of biomarkers have been proposed for early detection of AKI [3, 14]. In the present study, we deliberately focused on a marker of proximal tubular function in SA-AKI, and additional biomarkers beyond creatinine and FE_Na_ were therefore not analysed. Renal function in sepsis may be impaired in the absence of overt structural injury and may also vary substantially across similar degrees of structural injury; it is reasonable to assume that the same applies to tubular function [15]. The functional dimension captured by FE_Li_ should therefore, in future studies, be explored alongside histopathological changes, invasive physiological measurements, and established damage biomarkers in both experimental and human models.

In this study, samples were immediately frozen at − 70 °C and analysed as a single batch. However, same-day analysis and reporting are technically feasible and can be performed if prioritized and supported by sufficient laboratory resources. This highlights the potential for FE_Li_ to be implemented as a near-real-time functional biomarker in clinical practice.

## Conclusion

The results indicate an efficient proximal tubular sodium reabsorption in the non-AKI group, and significantly lower reabsorption in the SA-AKI group. All laboratory analyses in this study were performed in-house, highlighting feasibility of implementing FE_Li_ in clinical practise. Thus, measurement of endogenous lithium clearance may become a useful tool to monitor proximal tubular function in critically ill patients.

## Data Availability

Data are available from the corresponding author on reasonable request.

## Abbreviations

AKI: Acute kidney injury
C_Cr_, CrCl: Creatinine clearance
R_Naprox_: Estimated fractional proximal sodium reabsorption
eGFR: Estimated glomerular filtration rate
FE_Li_: Fractional excretion of lithium
FE_Na_: Fractional excretion of sodium
GFR: Glomerular filtration rate
ICP-MS: Inductively coupled plasma mass spectrometry
ICU: Intensive care unit
IQR: Interquartile ranges
KDIGO: Kidney disease: Improving global outcomes
C_Li_: Lithium clearance
MAP: Mean arterial pressure
RAAS: Renin-angiotensin-aldosterone system
SA-AKI: Sepsis-associated acute kidney injury
